# Investigating the role of GLUL as a survival factor in cellular adaptation to glutamine depletion *via* targeted stable isotope resolved metabolomics

**DOI:** 10.3389/fmolb.2022.859787

**Published:** 2022-08-12

**Authors:** Șafak Bayram, Yasmin Sophiya Razzaque, Sabrina Geisberger, Matthias Pietzke, Susanne Fürst, Carolina Vechiatto, Martin Forbes, Guido Mastrobuoni, Stefan Kempa

**Affiliations:** ^1^ Proteomics and Metabolomics Platform, Max-Delbrück-Center for Molecular Medicine (MDC), Berlin Institute for Medical Systems Biology (BIMSB), Berlin, Germany; ^2^ Mass Spectrometry Facility, Max Planck Institute for Molecular Genetics, Berlin, Germany; ^3^ Theoretical Chemistry Quantum Chemistry, Institute for Chemistry, Technische Universität Berlin, Berlin, Germany

**Keywords:** targeted stable isotope resolved metabolomics, GLUL, nucleotide biosynthesis, glutamine addiction, cancer metabolism, glutamine synthetase

## Abstract

Cellular glutamine synthesis is thought to be an important resistance factor in protecting cells from nutrient deprivation and may also contribute to drug resistance. The application of ‟targeted stable isotope resolved metabolomics” allowed to directly measure the activity of glutamine synthetase in the cell. With the help of this method, the fate of glutamine derived nitrogen within the biochemical network of the cells was traced. The application of stable isotope labelled substrates and analyses of isotope enrichment in metabolic intermediates allows the determination of metabolic activity and flux in biological systems. In our study we used stable isotope labelled substrates of glutamine synthetase to demonstrate its role in the starvation response of cancer cells. We applied ^13^C labelled glutamate and ^15^N labelled ammonium and determined the enrichment of both isotopes in glutamine and nucleotide species. Our results show that the metabolic compensatory pathways to overcome glutamine depletion depend on the ability to synthesise glutamine *via* glutamine synthetase. We demonstrate that the application of dual-isotope tracing can be used to address specific reactions within the biochemical network directly. Our study highlights the potential of concurrent isotope tracing methods in medical research.

## 1 Introduction

Reprogramming of cellular metabolism was the earliest molecular phenotypes described in cancer cells; Otto Warburg described the preference of cancer cells to ferment pyruvate into lactic acid even in the presence of oxygen and Hanahan and Weinberg finally included metabolic reprogramming into their list of hallmarks of cancer (Warburg, 1956; Vander Heiden et al., 2009; Hanahan and Weinberg, 2011)*.* Nowadays more and more detailed studies show a complex deregulation of cancer cell metabolism that is connected to growth and proliferation, immune cell evasion and also drug resistance mechanisms.

Despite a profound activation of glucose metabolism, cancer cells metabolise amino acids, such as glutamine (Lieu et al., 2020). Glutamine is a non-essential amino acid, and the most abundant in human blood plasma. Besides providing a source of energy, glutamine is required for several processes, including macromolecule biosynthesis, amino acid uptake, inhibition of autophagy and it triggers target of rapamycin (mTOR) kinase activation (Nicklin et al., 2009). Glutamine derived carbon fuels the tricarboxylic acid (TCA) cycle. The amino group of glutamine contributes to the synthesis of non-essential amino acids *via* the transaminase network and the amido-group serves as an obligate nitrogen donor for *de novo* nucleotide synthesis and hexosamine synthesis, specifically reactions that use the amido-nitrogen make glutamine a conditional essential metabolite (Altman et al., 2016; Bott et al., 2019).

Four different glutamine transport systems are characterized so far. These are known as SNAT3 (System N, SLC38A3) which is important in glutamine uptake in periportal cells in liver and in renal proximal tuble cells. SNAT1 (SLC38A1) is important in glutamine uptake by neuronal cells and ASCT2 (SLC1A5) is essential for glutamine uptake by rapidly growing epithelial cells and tumour cells in culture; the brush border membrane transporter B0 AT1 (SLC6A19) facilitates the uptake of glutamine across the kidney and intestinal brush border (McGivan and Bungard, 2007).

In the absence of sufficient extracellular glutamine, intracellular *de novo* synthesis can provide this essential metabolite. Glutamine synthetase (GS), also referred to as glutamate-ammonia ligase (GLUL) ligates glutamate with ammonia in an ATP-dependent condensation reaction (Nicklin et al., 2009). Several studies have revealed that the depletion of glutamine causes cell death (Eagle, 1955; Yuneva et al., 2007). This phenomenon, termed glutamine addiction, has been observed in a variety of cancer types in *in vitro* and *in vivo* studies (Wise and Thompson, 2011). Additionally, the reprogrammed metabolism of glutamine was shown to be crucial in tumorigenesis and tumour development (Yoo et al., 2020). Nevertheless, the molecular mechanisms underlying glutamine addiction are still not fully resolved.

Recent studies have demonstrated that glutamine deprived cells can be rescued by asparagine supplementation, for unclear reasons (Zhang et al., 2014). In the absence of glutamine, cells were rescued to a greater extent by asparagine supplementation relative to α-ketoglutarate, aspartate or glutamate (Zhu et al., 2017). In summary, asparagine has been demonstrated to regulate cell growth and rescue glutamine deficiency *via* several potential mechanisms (Zhang et al., 2014; Krall and Christofk, 2015; Zhang et al., 2017; Zhu et al., 2017; Pavlova et al., 2018). Understanding these mechanisms is fundamental to the development and efficacy of metabolic therapies targeting asparagine and glutamine metabolism. Interestingly rat stem cells transformed by the oncogenic Kaposi’s sarcoma-associated herpesvirus (KSHV) demonstrated the capacity to utilise the amido group from both glutamine and asparagine for purine and pyrimidine biosynthesis (Zhu et al., 2017). In our study we have shown that the ability of colon cancer cells to compensate glutamine withdrawal by asparagine supplementation did solely depend on intracellular *de novo* glutamine synthesis by glutamine synthetase (GLUL).

Dejure and Royla examined the growth behaviour of a panel of cell lines under glutamine supplemented and glutamine depleted conditions (Dejure et al., 2017). All tested colon cancer cells (HCT116, GEO, HT29, SW480, RKO) stopped proliferation in glutamine depleted conditions, while HEK293 cells were able to proliferate. Our investigations revealed that the ability of HEK293 cells to proliferate in glutamine deprived conditions was abolished when dialyzed serum was used in the growth medium. Therefore, the previously observed “glutamine independency” of HEK293 cells may be attributed to remaining small molecules enabling glutamine synthesis. To identify which amino acids enable cell growth in glutamine depleted conditions, cell growth assays were performed with supplementation of either glutamine, asparagine, glutamate, aspartate and alanine with or without ammonium. GLUL’s substrates, glutamate and ammonium, were associated with the greatest proliferation rate in the absence of glutamine in HEK293 and HCT116 cells. RKO cells were unable to proliferate in the absence of glutamine. The application of a competitive inhibitor of GLUL, methionine sulfoximine (MSO), prevented proliferation in the absence of glutamine also in HEK293 and HCT116 cells. Taken together, these findings pointed towards a key role for GLUL in adaptation to glutamine depletion.

To demonstrate GLUL activity and to determine the metabolic fate of GLUL’s substrates, a dual-tracer and targeted Stable Isotope Resolved Metabolomics (SIRM) method was established. We developed a “targeted SIRM” dual isotope tracing technique in which substrates specific to a biological reaction are differentially labelled and monitored *via* high resolution mass spectrometry. In this case, the simultaneous application of ^13^C-glutamate and ^15^N-ammonium allowed us to detect the relative contribution of extracellular glutamate and ammonium to intracellular glutamine synthesis, as well as monitor the downstream contribution of glutamine’s carbon and nitrogen to *de-novo* nucleotide biosynthesis. We also performed a time resolved dual-isotope tracing analysis and found the kinetics of glutamine synthesis in HEK293 and HCT116 cells are distinct, RKO cells did not show *de novo* glutamine synthesis, although the protein could be detected in proteomics analyses and western blot experiments.

Furthermore, we present growth conditions that preserve the viability of glutamine-dependent cancer cells under glutamine depletion. Our data show that all different amino acid supplementations that enable cell survival and proliferation with or without ammonium depend finally on the intracellular activity of GLUL. With the new established method of dual tracing and targeted pulsed stable isotope resolved metabolomics we could analyze the dynamics of intracellular glutamine synthesis.

## 2 Materials and methods

### 2.1 Cell culture

The standard cell culture medium (glutamine-supplemented medium) comprised Dulbecco’s Modified Eagle Medium (DMEM, Thermo Fisher) without glucose (Glc), glutamine (Gln), phenol red or sodium pyruvate, supplemented with 10% dialyzed fetal bovine serum (dFBS), 2.5 g/L Glc, and 2 mM Gln. HEK293, HCT116 and RKO cells were grown in 10 cm plates at 37°C, 5% CO_2_, 21% O_2,_ and 85% relative humidity, and were passaged every 3,4 days to avoid contact inhibition and supply new media. When a confluency of at least 70% was reached, cells were washed once with 1x PBS and detached from the plate *via* trypsinization with TrypLE (GIBCO). Pre-warmed medium was added to cease trypsinization. The volume of medium added was calculated according to the desired splitting ratio and the cells were resuspended before the appropriate fraction of the cell suspension was transferred to a new plate. For cell growth assays and subsequent experiments, cells were harvested at a confluency of 80%–90% before being transferred to new plates at a seeding density of 2×10^6^ cells which prevents contact inhibition.

### 2.2 Cell growth analysis

For the cell growth assays, pre-cultivated cells were seeded on 10 cm plates. The following day, the viable cell count was measured for the 0 hour time point and the cell culture medium was changed to that containing the appropriate condition (Gln: 2 mM; Alanine (Ala), Asparagine (Asn), Aspartate (Asp), Glutamate (Glu): all 1 mM, NH_4_^+^: 0.8 mM). Cells were passaged once they reached a confluency of at least 60%, upon which the cell count was determined. Media was replaced every 3,4 days to avoid limiting nutrients. Viability and cell number were monitored using the TC20 automated cell counter (Biorad).

### 2.3 Methionine sulfoximine inhibitor proliferation assay

Pre-cultivated cells were seeded on 6-well plates at a seeding density of 3×10^5^ cells and 12×10^5^ cells for HCT116 and HEK293 cells, respectively. The following day, the viable cell count was measured for the 0 hour time point and the cell culture medium was changed to that containing the appropriate culture condition (see Section 2.1) treated with either 500 µM Methionine Sulfoximine (MSO, Sigma Aldrich) or, as a negative control, sterile water (H_2_O). The viable cell count was determined at 24, 48, 72, and 96 h post-treatment. Media was replaced daily to replenish substrates and remove secreted reaction products.

### 2.4 Western blotting

Cells grown in standard media conditions (not starved for glutamine) were washed with PBS and harvested in 1 ml ice-cold RIPA buffer. Cell lysates of HCT116, RKO and HEK293 were denatured in loading buffer for 5 min at 95°C. 40 µg of proteins were loaded and separated on a 10% SDS gel and run for 1 h at 70 V and 1 h at 120 V. The gel was transferred to a nitrocellulose membrane (0.2 µm, Biorad) at 25 V, 1 A for 30 min (Biorad TransBlot Turbo V1.02). The membrane was blocked for 1.5 h in 5% milk in TBS-T at room temperature and cut below the 70 kDa band. The membranes were incubated with primary antibodies against Vinculin (1:2,000 dilution, Sigma, V9131) and against GLUL (1:1,000 dilution, Thermo Fisher, PA1-46165) in 5% milk in TBS-T over-night at 4°C. After washing the membranes in TBST, the membranes were incubated in the HRP-conjugated secondary antibodies (NEB, 7074S; NEB, 7076S) for 1 h at room temperature. After washing the membranes in TBST and TBS, the membrane was developed using an ECL Western Blotting detection reagent (Amersham, RPN2109) according to the manufacturer’s protocol. The Vilber FX gel system was used to record the luminescence (Vilber Lourmat, France).

### 2.5 Targeted stable isotope resolved metabolomics and pSIRM

Cells were pre-cultivated in Glu + NH_4_^+^-supplemented medium for at least 3 days prior to stable SIRM analysis. HEK293 and HCT116 cells were able to proliferate in Glu + NH_4_^+^-supplemented dFBS medium and were therefore pre-cultivated for over one month for cell growth assays before being seeded at a density of 2E+6 cells on 10 cm plates. RKO cells were unable to proliferate in Glu + NH_4_^+^-supplemented medium and were therefore pre-cultivated in Gln-supplemented dFBS medium and changed to medium containing Glu + NH_4_^+^ performed 3 days prior to the SIRM experiment.

For SIRM experiments, cells were then labelled for 24 h with ^13^C labelled glutamate and ^15^N labelled ammonium and treated in parallel with either 500 µM Methionine Sulfoximine (MSO, Sigma Aldrich) or, as a negative control, sterile water (H_2_O). For pSIRM experiments, cells were pre-treated for 6 h with either 1 mM MSO or, as a negative control, sterile water, in fresh Glu + NH_4_^+^-supplemented dFBS medium. Afterwards, cells were labelled for 15 min, 30 min, 1 h, and 3 h with ^13^C labelled glutamate and ^15^N labelled ammonium, with or without 1 mM MSO. In both experiments, SIRM and pSIRM, 1 mM ^13^C labelled glutamate was used. However, for SIRM experiments 96 µM ^15^N labelled ammonium were used, whereas for pSIRM 0.8 mM ^15^N labelled ammonium were used. Cells were harvested and extracted in a methanol-chloroform-water solution as described elsewhere [DOI: 10.1016/B978-0-12-801329-8.00009-X].

Intracellular amino acids were measured as TBDMS derivatives by high-resolution GC-MS. Dried cellular extracts were mixed with 25 µl MTBSTFA (Sigma) and 25 µl ACN and incubated at constant shaking for 1 h at 80°C. Derivatization was automatized on a TriPlus RSH auto-sampler (Thermo Fisher) and each sample was injected immediately after the derivatization. Samples were injected into a Q Exactive GC Orbitrap system (Thermo Fisher) with a splitof 1:5 (1 µl injection volume) in a temperature-controlled injector (TriPlus RSH auto-sampler, Thermo Fisher) with a baffled glass liner. The initial temperature was 80°C for 15 s, followed by an increase of 7°C/s up to 260°C, which is held for 3 min at the end of the temperature program. Gas chromatographic separation was carried out on a Trace 1,300 GC (Thermo Fisher) equipped with a TG-5SILMS column (30 m length, 250 µm inner diameter, 0.25 µm film thickness (Thermo Fisher). Helium was used as the carrier gas (1.2 ml/min flow rate). Gas chromatography was performed with an initial temperature of 68°C for 2 min, followed by an increase of 5°C/min up to 120°C, followed by an increase of 7°C/min up to 200°C, followed by an increase of 12°C/min up to 320°C which is held for 6 min. The spectra were recorded in a mass range of m/z = 60––600 with resolution at 200 m/z set at 120,000.

The elemental composition of different fragments for glutamine were calculated based on the exact mass and compared with known literature-values. To extract the intensities for the different isotopic masses we constructed a compound library including the mass shifts induced by ^13^C and ^15^N. Mass shifts were calculated *via* a custom R-Script based on the known masses for the fragments and the number of potentially incorporated carbon and nitrogen atoms. Each incorporated ^13^C or ^15^N increased the target mass by 1.0033548 or 0.99693689, respectively.

For the SIRM experiment, samples were then processed and peaks were integrated with Tracefinder 5.0 (Thermo Fisher), by importing this target list as a Tracefinder Compound database and extracting the extracted ion chromatograms (EIC) within a 5 ppm window. For the pSIRM experiment, samples were processed and peaks integrated in Xcalibur Quanbrowser (Thermo Fisher), extracting EIC with a mass tolerance of 2.5 ppm. For both, peak integration quality was visually checked and finally all peak areas were exported.

### 2.6 Measurement of free nucleotides

Free nucleotides were measured by direct infusion MS on a Q Exactive HF (Thermo Fisher) coupled to a Triversa Nanomate (Advion) nanoESI ion source. The Triversa Nanomate was operated in negative mode, with 1.5 kV spray voltage and 0.5 psi head gas pressure. The spectra were recorded for a duration of 3 min in a mass range of m/z = 140–850 m/z mass units with resolution at 200 m/z set at 240,000. A target list with 48 compounds was prepared in a similar way as described above. The M-H fragment was further calculated by subtracting 1.00728 from the exact mass of the uncharged molecule. For the extraction of the peak intensities the raw files were first converted to.dta2d files using TOPPAS FileConverter tool (Kohlbacher et al., 2007a; Kohlbacher et al., 2007b; Sturm et al., 2008).

The.dta2d files were then processed with a custom R script. Briefly, zero intensities and TIC intensities were removed from the datafiles as well the first and last five scans as these scans tend to be instable. All masses that fit into a 5 ppm window for each mass in the target list was associated to that specific compound. To extract only the apex the most intense mass per compound and scan was kept. Finally, the median and the standard deviation for all the scans was calculated to obtain a single readout per compound and sample.

Natural abundance correction for both types of experiment was performed using the Accucor package (URL: https://doi.org/10.1021/acs.analchem.7b00396).

## 3 Results

### 3.1 Cell growth assay in fetal bovine serum vs. dialyzed fetal bovine serum

Dejure and Royla, tested the effect of glutamine starvation on GEO, HCT116, HEK293, HT29, RKO and SW480 cells and found that all cell lines were not able to proliferate except HEK293 (Dejure et al., 2017).

**FIGURE 1:**
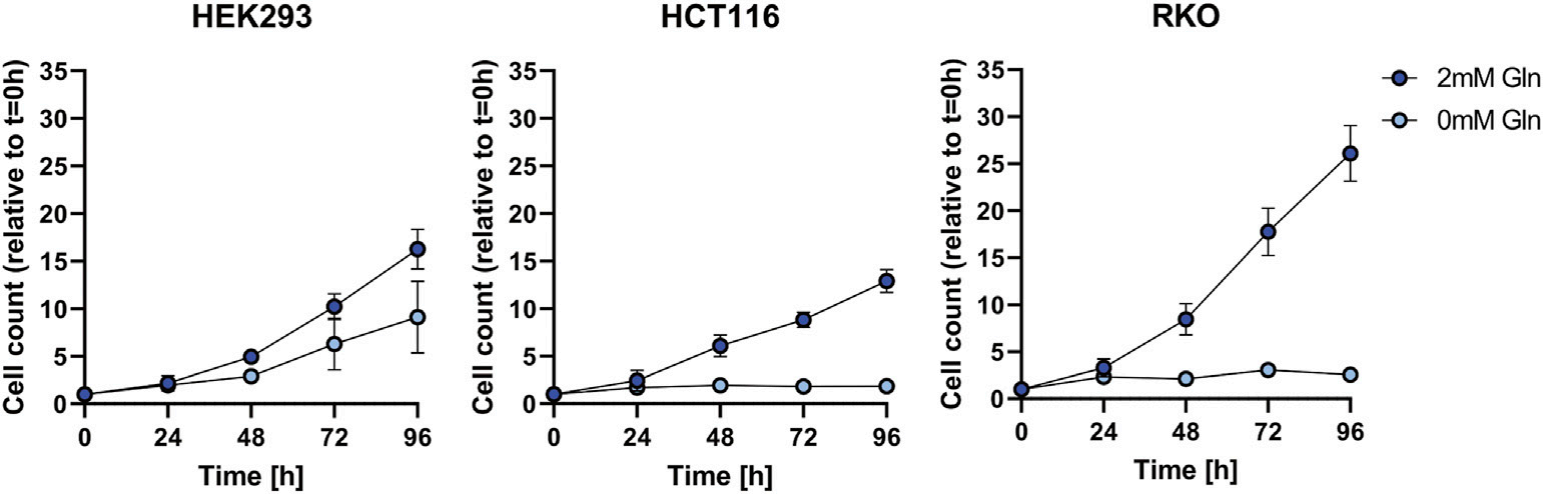
Cell growth assay for HEK293, HCT116, and RKO cells in non-dialyzed FBS with 2 mM or 0 mM glutamine (Gln) in the cell culture media. Cell count was determined every 24 h over the course of 96 h and is shown relative to *t* = 0 as mean ± SD.

**FIGURE 2:**
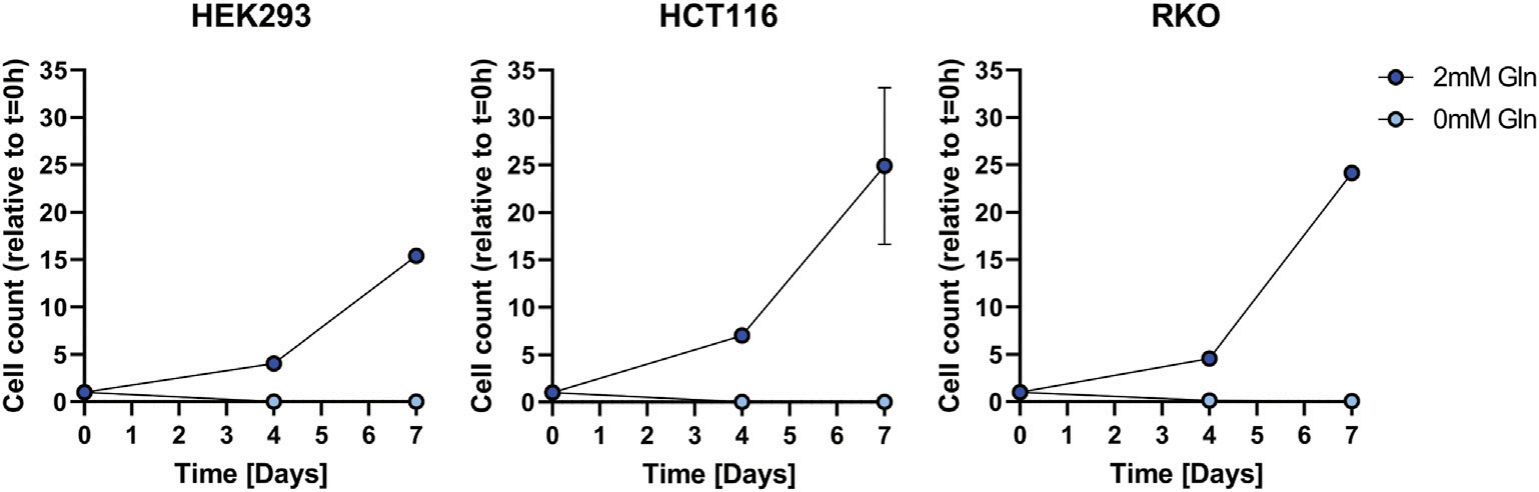
Cell growth assay for HEK293, HCT116, and RKO cells in dialyzed FBS with 2 mM or 0 mM glutamine (Gln) in the cell culture media. Cell count was determined over the course of 7 days and is shown relative to *t* = 0 as mean ± SD.

We investigated as to whether two colon cancer cell lines HCT116 and RKO can can adapt to glutamine depletion and removed glutamine from the medium for several days (Figure 1). Also in this experiment HEK293 cells exhibited glutamine independence, but RKO and HCT116 cells were not able to proliferate. In order to exclude that the glutamine independence of HEK293 cells is not caused by small molecules provided by the fetal bovine serum (FBS) we used dialyzed FBS and repeated the proliferation experiment (Figure 2). Interestingly, in this case also HEK293 cells were not able to proliferate when glutamine was deprived. Thus, glutamine was also essential for HEK293 cells when using dialyzed FBS.

### 3.2 Cell growth assay in supplemented dialyzed fetal bovine serum

**FIGURE 3:**
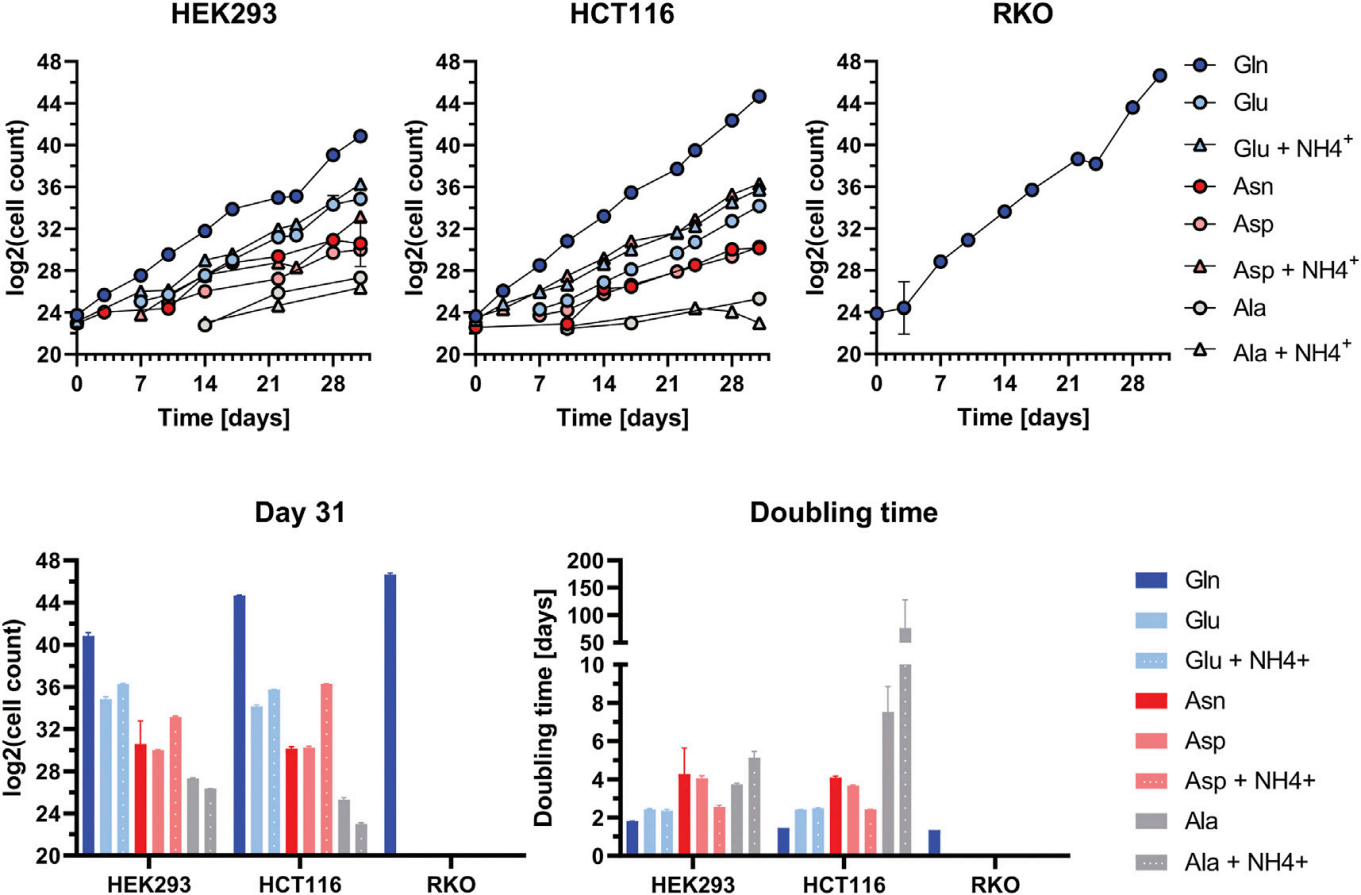
Cell Growth Assay in supplemented dialyzed FBS in HEK293, HCT116, and RKO cells. Cell growth upon the application of various amino acid substrates: Alanine (Ala): 1 mM; Ala: 1 mM + NH_4_^+^: 0.8 mM; Asparagine (Asn): 1 mM; Aspartate (Asp): 1 mM; Asp: 1 mM + NH_4_^+^: 0.8 mM; Glutamate (Glu): 1 mM; Glu: 1 mM + NH_4_^+^: 0.8 mM; Glutamine (Gln): 2 mM. Viable cell count was determined at every passage over the course of 31 days. Cell count data (each *n* = 2) were Log2-transformed and are shown as mean ± SD. The doubling time was calculated based on the duration in culture and the number of duplications underwent during this time (i.e., duration/Δlog2(cell count)).

In order to identify which metabolic pathways are efficiently utilised in glutamine-depleted condition, we monitored cell survival and growth upon supplementation with substrates of the glutamine-centric metabolic network. To achieve this, we supplemented: Alanine (Ala), Ala + ammonium (NH_4_^+^), Asparagine (Asn), Aspartate (Asp), Asp + NH_4_^+^, Glutamate (Glu), Glu + NH_4_^+^ in dialyzed FBS medium (Figure 3).

Viable cell count was determined at every passage over the course of 31 days. Cell count data were log2-transformed and graphically represented. Cell doubling time was calculated based on the division of culture duration by delta in log2-transformed cell counts. All three tested cell lines exhibit the highest proliferation rate and lowest doubling time in Gln-supplemented medium (Figure 3). In HEK293 and HCT116 cells the proliferation rate in Glu + NH_4_^+^- supplemented medium is close to that in Gln-supplemented medium. For HCT116 cells proliferation in Asp + NH_4_^+^-supplemented media is remarkably high. In HEK293 cells also the addition of glutamate leads to intermediate cell proliferation rates. Contrary, RKO cells can not compensate glutamine withdrawal under any condition. RKO cell viability decreased and cell death occurred, preventing the possibility of obtaining viable cell count data after 5 days onwards. Therefore, the proliferation rate for Ala, Ala + NH_4_^+^, Asn, Asp- Asp + NH_4_^+^, Glu, Glu + NH_4_^+^-supplemented media is 0.

### 3.3 Methionine sulfoximine inhibitor proliferation assay

The cell growth assays show that in glutamine-depleted dialyzed FBS conditions, HEK293 and HCT116 cells proliferate best when supplemented with the substrates of GLUL: Glu and NH_4_^+^(Figure 3). Based on this result, an inhibitor assay was performed to assess the effect of blocking *de novo* glutamine synthesis. Therefore, cells were treated with MSO, a competitive inhibitor of GLUL. As RKO cells are unable to proliferate in glutamine-depleted conditions, they were not subjected to this assay.

**FIGURE 4:**
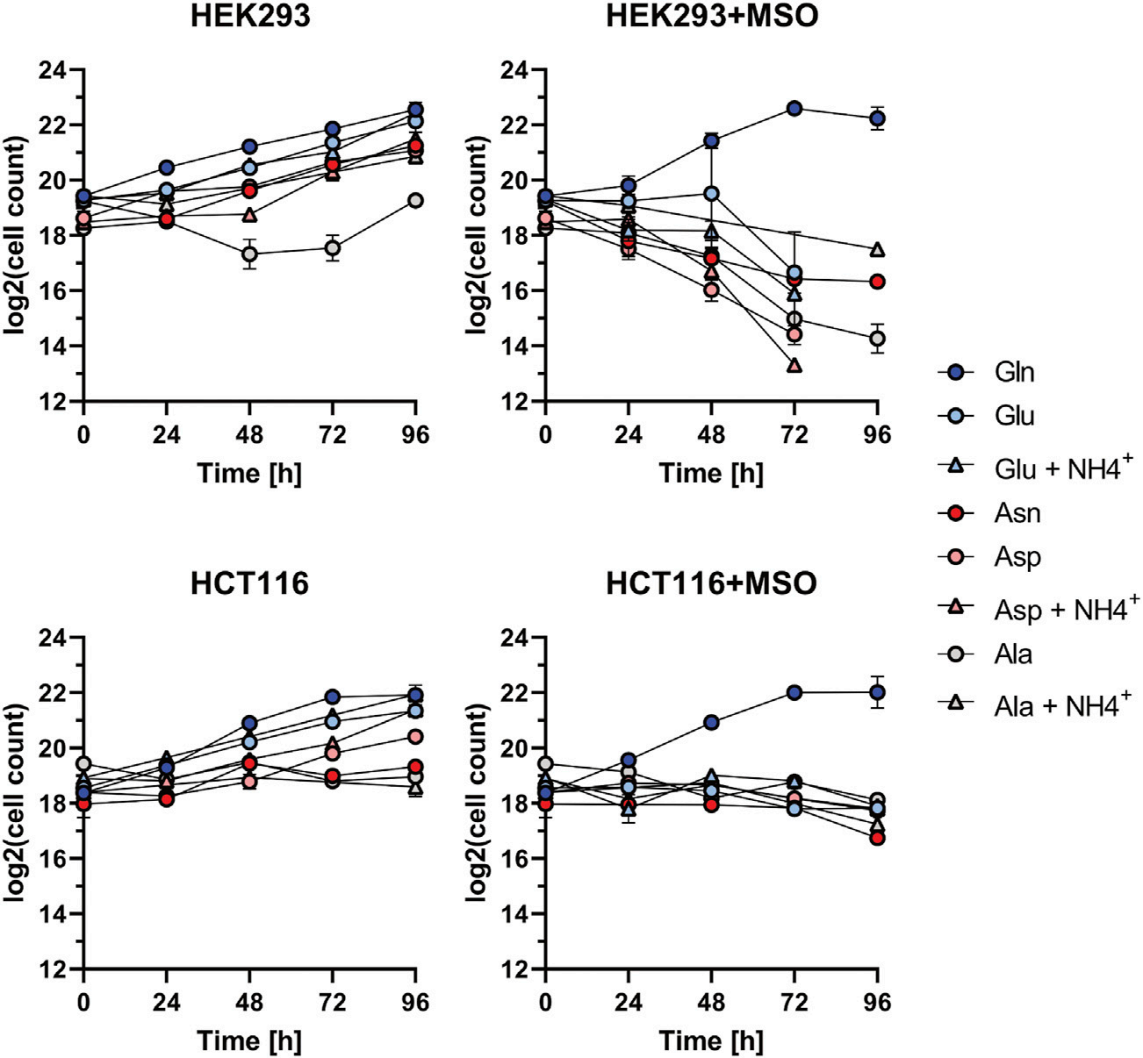
Proliferation inhibition assay for HEK293, HCT116, and RKO cells. Investigation of cell growth upon the application of GLUL inhibitor MSO (500 µM) or H_2_O with Alanine (Ala): 1 mM; Ala: 1 mM + NH_4_^+^: 0.8 mM; Asparagine (Asn): 1 mM; Aspartate (Asp): 1 mM; Asp: 1 mM + NH_4_^+^: 0.8mM; Glutamate (Glu): 1mM; Glu: 1 mM + NH_4_^+^: 0.8 mM; Glutamine (Gln): 2 mM. Cell count (each *n* = 2) was determined every 24 h over the course of 96 h and is shown relative to *t* = 0 as mean ± SD.

A pilot experiment (data not shown) was performed in HEK293 and HCT116 cells and an inhibitor concentration of 500 µM was found to be effective. The inhibitor-containing medium was refreshed and viable cell count was determined every 24 h over a 96 h time period. A parallel assay was performed using water, the solvent control, instead of MSO. Each measurement was taken from three biological replicates. The mean and standard deviation of the viable cell counts for each time point were graphically represented (Figure 4). Untreated HEK293 and HCT116 cells exhibit similar proliferation rates to those observed in the previous growth assay for all tested different conditions. However, MSO-treated HEK293 and HCT116 cells only show proliferation in Gln-supplemented medium. Showing that the chosen inhibitor concentration is not harmful to cells if glutamine is provided.

### 3.4 Targeted stable isotope resolved metabolomics and methionine sulfoximine-treatment

We designed a dual tracer stable isotope resolved metabolomics (SIRM) study using ^13^C and ^15^N labelled substrates (^13^C glutamate, ^15^N ammonium) to determine whether the cells utilise extracellular substrates for *de novo* glutamine synthesis and subsequent nucleotide biosynthesis. Based on the results of the cell growth assays, Glu + NH_4_^+^-supplemented medium was chosen to trace nitrogen and carbon incorporation into glutamine. The experiment was performed in three biological replicates.

Glutamine can be synthesised by GLUL-mediated ligation of glutamate and ammonium, with ammonium providing the amido-group. The ligation of ^13^C_5_-glutamate and ^15^N-ammonium was monitored by GC-MS detection of ^13^C_5_-, ^14^N_1_-, and ^13^C_5_- ^15^N_1_-glutamine isotopologues. Automated peak extraction from GC-MS spectra was performed *via* Tracefinder (Thermo Fisher) and mean ^13^C and ^15^N enrichment calculations were performed using custom R scripts. In HCT116 and HEK293 cells, ^13^C- and ^15^N-incorporation into glutamine was detected in several characteristic glutamine-3TBDMS fragments, as indicated by the corresponding ^13^C- and ^15^N-induced mass shift of the peaks. Glutamine-3TBDMS fragments comprising a 5C skeleton and 2N atoms underwent a mass shift of approximately 6 Da (m+6) while fragments comprising a 4C skeleton and 2N atoms underwent a mass shift of 5 Da (m+5), corresponding to ^13^C_5_-^15^N_1_ and ^13^C_4_- ^15^N_1_ isotopologues, respectively. The cleanest signal was obtained for the fragment at 431 m/z and therefore this fragment was used for further analysis.

**FIGURE 5:**
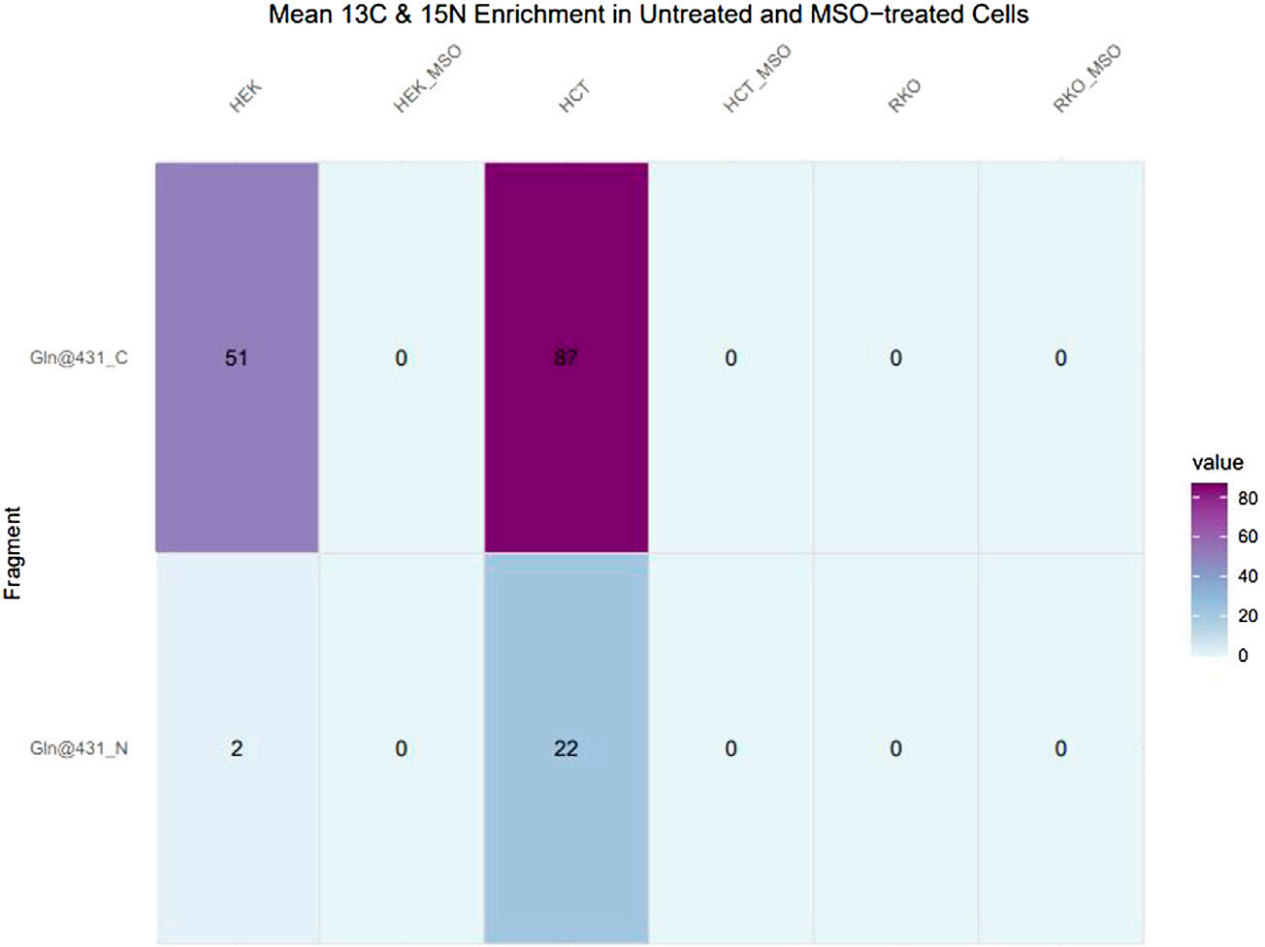
^13^C and ^15^N enrichment in glutamine in untreated and MSO-treated HEK293, HCT116 and RKO Cells. Cells were cultivated with ^13^C5-glutamate and ^15^N-ammonium for 24 h. After obtaining mass spectra, peak areas were extracted and natural isotope abundance correction and isotope enrichment calculations were performed. Data represent mean ^13^C and ^15^N enrichment (%).

In the pilot experiment HCT116 and HEK293 cells were labelled for 24 h with 13C glutamate and 15N ammonium. In HEK293 cells 51% enrichment of ^13^C and 2% enrichment of ^15^N in the glutamine-3TBDMS fragment were monitored. HCT116 cells have almost twice as much ^13^C enrichment at 87% and ^15^N enrichment at 22%. In both HEK293 and HCT116 cells MSO treatment abolished ^13^C and ^15^N enrichment to 0%. RKO cells do not demonstrate ^13^C or ^15^N enrichment in untreated and MSO-treated conditions (Figure 5).

**FIGURE 6:**
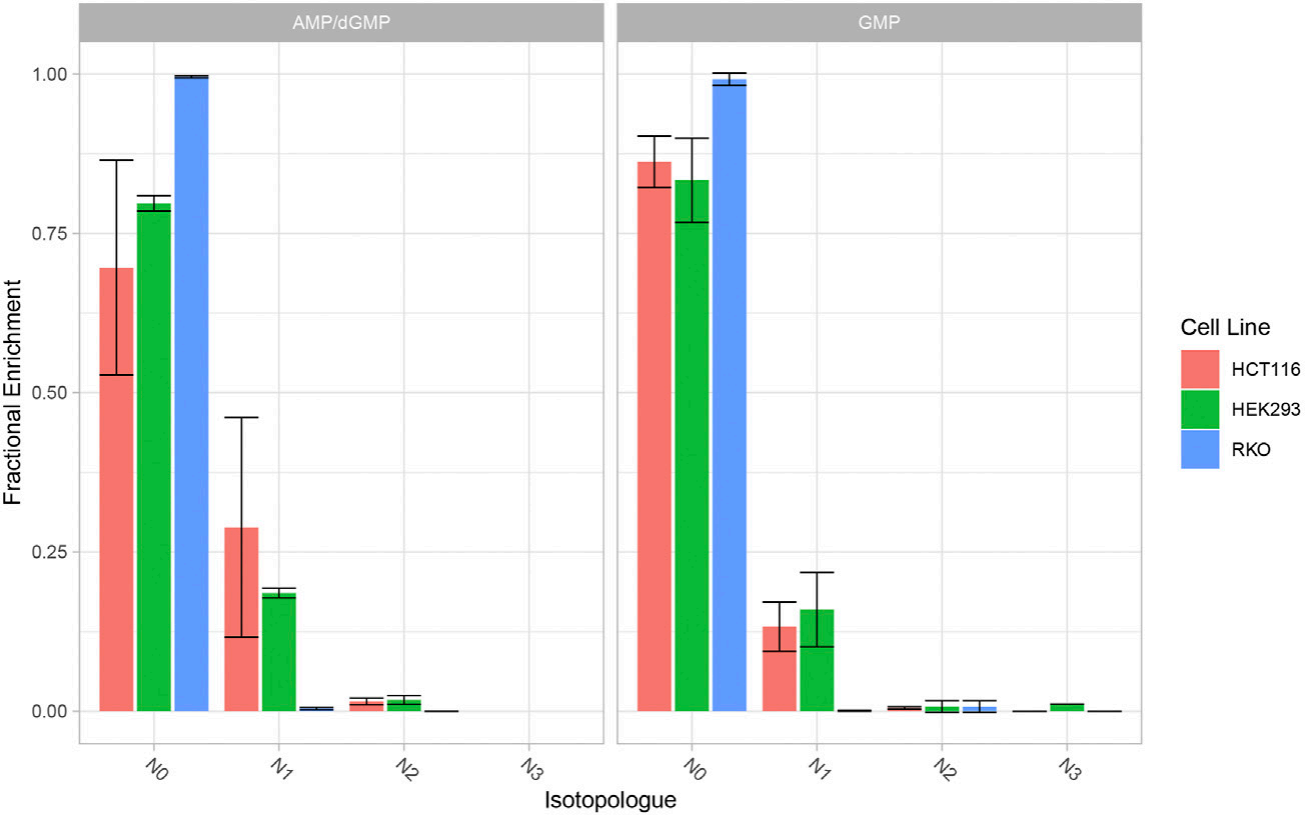
^15^N Enrichment in AMP/GMP (purine nucleotides) in HEK293, HCT116 and RKO cells. Cells were cultivated with ^13^C_5_-glutamate and ^15^N-ammonium for 24 h. Nucleotide isotopologues were measured *via* direct-infusion MS and relative quantities are graphically represented. Data represent mean ± SD of three biological replicates.

The contribution of newly synthesised glutamine isotopologues to nucleotide biosynthesis was monitored by direct-infusion MS detection of nucleotide isotopologues. Peak extraction from direct infusion-MS spectra was performed manually with XCalibur Qualbrowser (Thermo Fisher). In the *de novo* purine biosynthesis pathway, glutamine donates two nitrogen atoms to IMP and AMP, and three nitrogen atoms to GMP. HEK293 and HCT116 demonstrate ^15^N enrichment in AMP and GMP while RKO cells do not. In HEK293 and HCT116 cells, one ^15^N atom (N1) is incorporated into each AMP and GMP. HEK293 cells exhibit 25% enrichment of ^15^N_1_-AMP and 20% enrichment of ^15^N_1_-GMP, while HCT116 cells exhibit 35% enrichment of ^15^N_1_-AMP and 15% enrichment of ^15^N_1_GMP (Figure 6).

**FIGURE 7:**
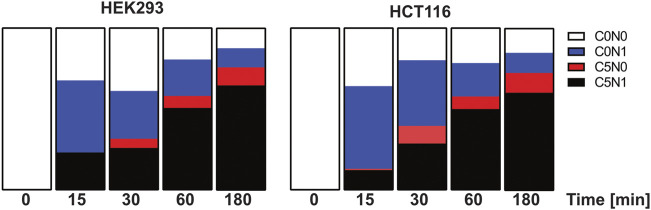
Isotope incorporation into glutamine after pulse labelling with ^13^C_5_-glutamate and ^15^N-ammonium in HCT116 and HEK293 cells. HCT116 and HEK293 cells were incubated with ^13^C_5_-glutamate and ^15^N-ammonium for 15 min, 30 min, 1 h, and 3 h (*n* = 2 each). Shown are the relative pool sizes of non-labelled (C0N0), ^15^N labelled (C0N1), ^13^C_5_ labelled (C5N0) ^15^N-^13^C_5_ labelled (C5N1) glutamine.

In a second step we analyzed the dynamics of glutamine synthesis in HCT116 and HEK293 cells in a time course manner. The cell lines were incubated for 15 min, 30 min 1 h and 3 h with ^13^C labeled glutamate and ^15^N labeled ammonium. Label incorporation in glutamine was analyzed as described above (Figure 7). Interestingly label incorporation in glutamine can be found already after 15 min in HCT116 cells and after 30 min in HEK293 cells. Both cell lines show a fast glutamine synthesis but the kinetics are different. In HCT116 cells the incorporation of ^15^N labeled ammonium into glutamine exceeds the formation of carbon and nitrogen labeled glutamine, this argues for faster ammonium import into HCT116 cells compared to HEK293 cells.

Interestingly, we performed a western blot analysis to analyze GLUL protein expression in the three cell lines and found that GLUL protein is present in all cell line even under normal conditions (Supplementary Material). We compared the western blot result with proteomics data (not shown) and could also find specific peptides for GLUL in all cell lines. Thus, the reason for the lacking GLUL activity in RKO cells cannot be explained by missing GLUL protein levels but must be caused by other reasons, e.g., mutations in the GLUL gene or impaired transport of substrates for GLUL reaction.

## 4 Discussion

So far stable isotope tracing studies with multiple isotopic tracers were performed without specific applications to demonstrate how this technology can add more information, compared to single isotope tracing methods. Here we show for the first time that this technology can be used to address specific reactions in the metabolic network and to address clinically relevant questions. In our study we analyzed the activity of glutamine synthetase (GLUL) by applying both substrates glutamate and ammonium labelled with stable isotopes.

Glutamine synthetase (GLUL), is of major interest, because this enzyme may be a resistance factor in metabolic cancer treatments; like the asparaginase treatment for acute lymphoblastic leukemia (ALL) and solid cancer (Rotoli et al., 2005). Glutamine is an important nutrient supporting cell growth and proliferation, oncogenic mutations often render cancer cells glutamine-dependent (Altman et al., 2016). In glutamine-depleted conditions, α-ketoglutarate, aspartate and glutamate supplementation have been demonstrated to rescue cell growth of glutamine-dependent cancer cells to a certain extend (Zhu et al., 2017).

In our study we analyzed the nature of glutamine addiction of three selected cell lines. HEK293 cells were partially glutamine auxotroph and HCT116 and RKO cells glutamine addicted (Dejure et al., 2017). We found that the ability of HEK293 cells to proliferate under glutamine deprived conditions did depend on the usage of non-dialyzed FBS, if we used dialyzed serum also HEK293 cells did depend on external glutamine supply. This demonstrates that glutamine addiction is found in all tested cell lines in our study.

**FIGURE 8:**
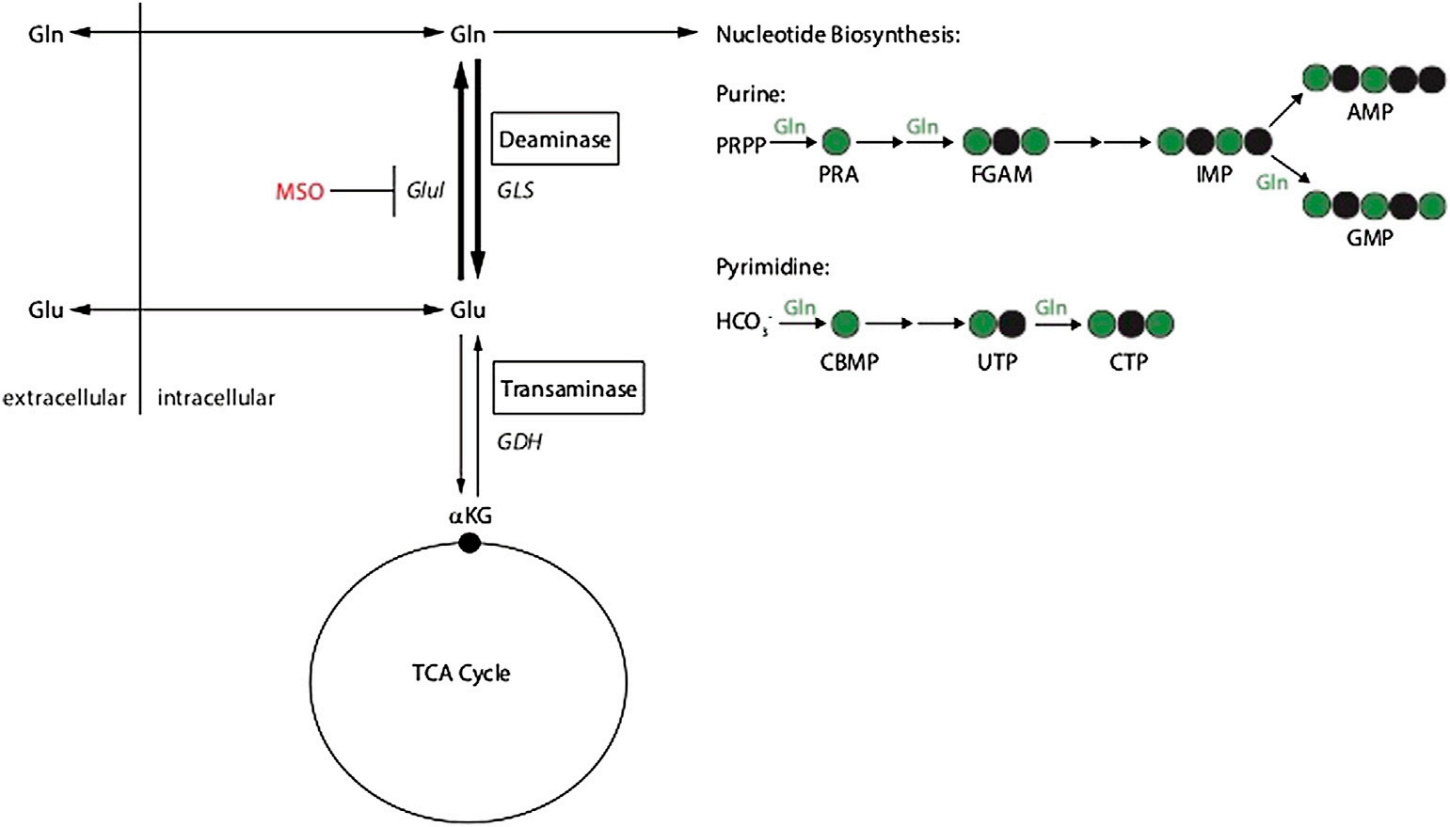
Schematic of glutamine metabolism and MSO inhibition of glutamine synthetase (GLUL).

In order to investigate the metabolic pathways that can contribute to glutamine autotrophy we applied a selected set of amino acids in a defined knock out medium. Although these conditions are artificial or synthetic compared to the natural environment of a cancer cell, this experiment can be used to understand the metabolic wires around glutamine (Figure 8); we supplemented: Alanine (Ala), Ala + ammonium (NH4+), Asparagine (Asn), Aspartate (Asp), Asp + NH4+, Glutamate (Glu), Glu + NH4+ in dialyzed FBS medium. Because of the absence of GLUL activity in RKO cells none of the supplements could contribute to cell growth and survival. This result clearly shows that all pathways that allow glutamine independency funnel into *de-novo* glutamine synthesis *via* GLUL. Similarly, the application of the specific GLUL inhibitor MSO abolished the capacity of HEK293 and HCT116 cells to proliferate without glutamine using the supplemented nutrients. Our results demonstrate once more that GLUL is the major player in glutamine independence.

Using high resolution mass spectrometry, we were able to monitor GLUL activity *via* a dual-tracer targeted SIRM approach. This method allowed us to measure a specified reaction by the application of multiple isotopic tracers. We observed in the pilot experiment a cell-line specific incorporation of extracellular ammonium into glutamine: HCT116 cells displayed a higher incorporation of extracellular ammonium for glutamine synthesis than HEK293 cells. However, we do not know if the available ammonium is spent within 24 h. In subsequent experiments the ammonium concentration was increased. In order to retrieve dynamic information about the uptake rates of individual substrates, a time course experiment was performed. The time and stable isotope resolved metabolomics experiments using multiple tracers delivered valuable information about the metabolic activity facilitated in the cell lines. The data show that HCT116 cells have a faster uptake of glutamate and that internal ammonium is used within the first 15 min of the pulse experiment. The data indicate that HCT116 cells possess higher glutamine synthesis rates. Both cell lines show high levels of stable isotope enrichment in glutamine after 30 min labeling time.

By using direct-infusion MS, we detected ^13^C and ^15^N enrichment in the *de novo* purine biosynthesis pathway in HEK293 and HCT116 cells, when ^13^C glutamate and ^15^N ammonium were supplied. To demonstrate the essentiality of GLUL activity to *de novo* glutamine synthesis and downstream nucleotide synthesis, inhibition of GLUL *via* MSO treatment ablated ^13^C and ^15^N incorporation.

Interestingly, RKO cells do not demonstrate ^13^C and ^15^N incorporation even in the absence of MSO treatment, indicating that either GLUL is inactive or the import of these substrates is compromised. The results from the dual-tracer targeted SIRM study reflect the observations from the cell growth and inhibitor assays (Figure 6). Taken together; the cell growth assays, inhibitor studies and SIRM analyses reveal that, in glutamine depleted conditions cell growth is dependent upon *de novo* glutamine synthesis.

The usage of the dual isotope tracing strategy to measure targeted and enzymatic activity in the cellular network in a time resolved manner is an advantage. This was not done so far, and our study is the first showing the power of this technology also for a clinically relevant question. We could show at multiple layers that, despite glutamine synthetases is expressed at the protein level, GLUL activity is absent in RKO cells, thus we show that expression levels alone cannot explain all metabolic activities.

The complex growth experiment highlights the role of an active glutamine synthesis to rescue glutamine withdrawal by using other amino acids, or a combination of amino acids and ammonium. We could also show that in all the reactions that we tested GLUL is the key enzyme and consequently; blocking glutamine synthetase with the inhibitor MSO abolishess growth and proliferation in the tested cell lines. Therefore, we propose that this method can be applied in clinical studies assessing different kinds of tumour cells and measuring glutamine synthetase activity *in vivo*.

Overall, we show that concurrent stable isotope labelling serves as a powerful tool for probing not only metabolic pathways, but also independent enzymatic reactions. Leveraging this tool enabled us to validate our observations from *in vitro* cell-based assays and demonstrate an essential reaction underlying the capacity of cells to adapt to glutamine-depletion. However, to detect mass shifts induced by small molecules such as ^13^C and ^15^N atoms, a very high resolution is needed (Su et al., 2017). In the absence of such a high resolution, mathematical models can be used to calculate the relative contribution of these molecules. We utilised the R package IsoCorrectoR to calculate the relative contribution of ^13^C and ^15^N in glutamine. For the purine nucleotides, we were able to resolve ^13^C and ^15^N incorporation from the raw data.

Our dual-tracer targeted SIRM study highlights the potential for high resolution mass spectrometry to monitor specific biological reactions at the atomic level. In future it can be envisioned to study more enzymatic reactions using concurrent isotope tracing techniques. We propose that all metabolic reactions that require two or more substrates that can be addressed with diverse isotopic labeling can be analyzed using this method, e.g., reactions within the *de novo* nucleotide biosynthesis or hexose amine biosynthesis. This will be of mayor advantage if enzymatic activity essays are not established.

## Data Availability

The raw data supporting the conclusion of this article will be made available by the authors, without undue reservation.
