# Supplementary figures and images for: Investigating the role of GLUL as a survival factor in cellular adaptation to glutamine depletion via targeted stable isotope resolved metabolomics
Source: Front Mol Biosci. 2022 Aug 12;9:859787. doi: 10.3389/fmolb.2022.859787 (PMC9412915; doi:10.3389/fmolb.2022.859787)

Supplemental information:

Supplemental figure 1. Wester blot of GLUL in HCT116, HEK293 and RKO cells.


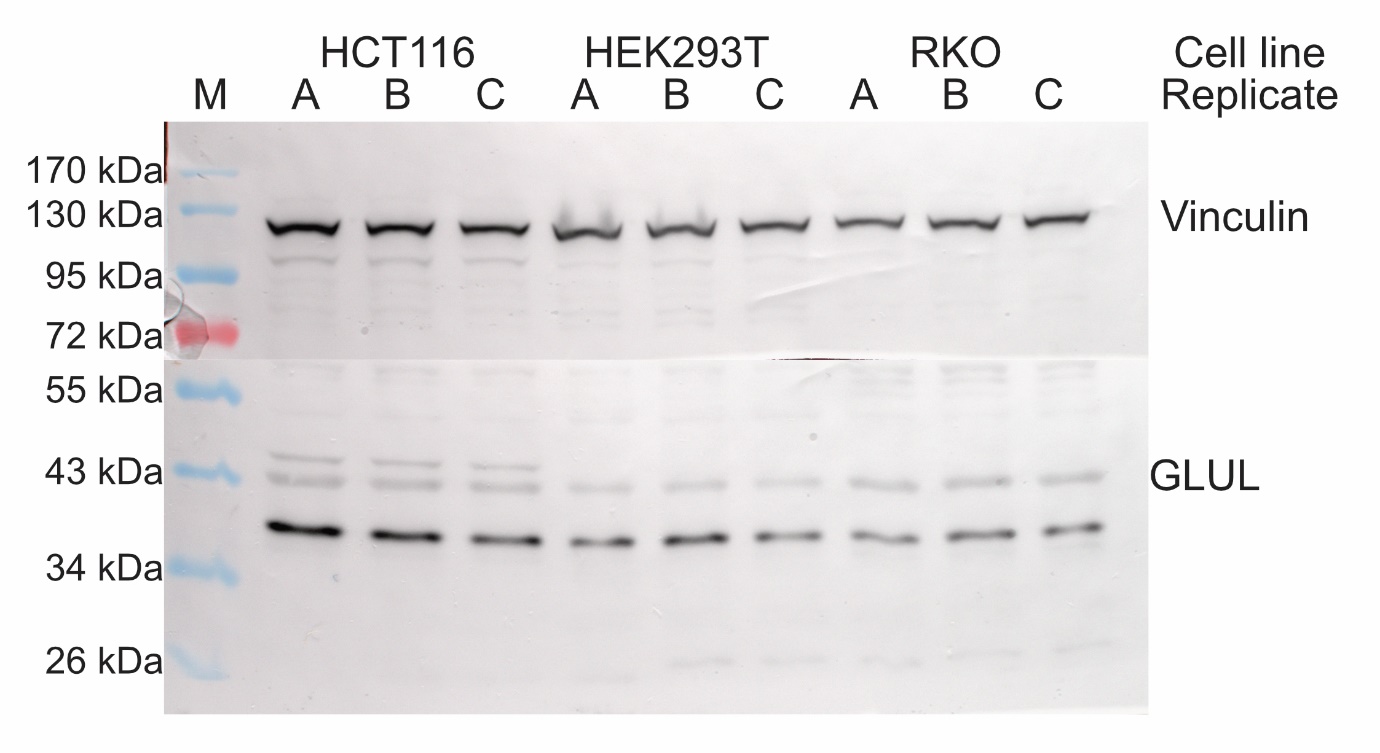

Supplement: Supplementary file 1 [file Table1.DOCX]
